# Systemic Sarcoidosis Presenting in a Scar

**DOI:** 10.1155/2023/7751754

**Published:** 2023-01-24

**Authors:** Amy Xiao, Lauryn M. Falcone, Joseph C. English III

**Affiliations:** ^1^School of Medicine, University of Pittsburgh, Pittsburgh, PA, USA; ^2^Department of Dermatology, University of Pittsburgh, Pittsburgh, PA, USA

## Abstract

While most forms of sarcoidosis of the skin do not require treatment, 40% of patients initially diagnosed with cutaneous sarcoidosis are found to have an asymptomatic disease involving other organ systems. It is the involvement of the lungs, heart, eyes, and nervous system which most often contributes to morbidity/mortality. An early and accurate diagnosis of sarcoidosis is difficult because patients may be asymptomatic, initial presentations may vary, and there is no single reliable diagnostic test except biopsy. We present a case of scar sarcoidosis which led to the diagnosis of stage II pulmonary sarcoidosis in a woman in her 50s. Her scar sarcoidosis presented as well-circumscribed, reddish-brown macules surrounding an atrophic scar from a prior skin graft on the right leg. Biopsy revealed scattered, well-formed, non-necrotizing granulomas of the dermis composed of epithelioid histiocytes and multinucleated giant cells, surrounded by a sparse infiltrate of lymphocytes and histiocytes. A CT chest demonstrated extensive hilar lymphadenopathy, leading to a diagnosis of stage II pulmonary sarcoidosis with cutaneous involvement. This case illustrates the interesting presentation of scar sarcoidosis and underscores the importance of a broad differential including sarcoidosis for skin changes around scars and underscores the need for early biopsy. Prompt cutaneous diagnosis leads to earlier systemic evaluation, therapeutics, and better outcomes.

## 1. Introduction

While most forms of sarcoidosis of the skin are mild and do not require treatment, approximately 40% of patients initially diagnosed with cutaneous sarcoidosis are found to have asymptomatic disease involving other organ systems [1, 2]. Furthermore, it is the involvement of the lungs, heart, and nervous system which most often contributes to mortality from this disease [3]. However, an early and accurate diagnosis of sarcoidosis is difficult because patients may be asymptomatic, initial presentations may vary, and there is no single reliable diagnostic test [4]. Earlier diagnosis and treatment of pulmonary sarcoidosis lead to better outcomes for the patient, as increasing stage at diagnosis is correlated with decreasing rates of spontaneous resolution [4].

## 2. Case Study

**Figure 1:**
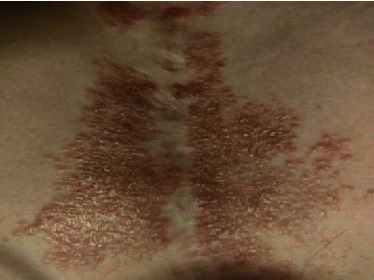
A scaly, erythematous patch on the anterior chest surrounding a previous surgical scar.

**Figure 2:**
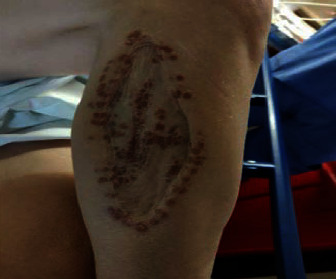
Well-circumscribed, reddish-brown, slightly scaly macules surrounding a large atrophic scar figure from a prior skin graft on the right leg.

**Figure 3:**
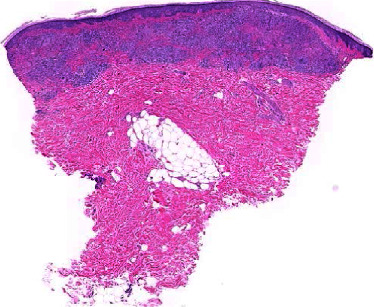
H&E-stained biopsy specimen at 10x magnification.

**Figure 4:**
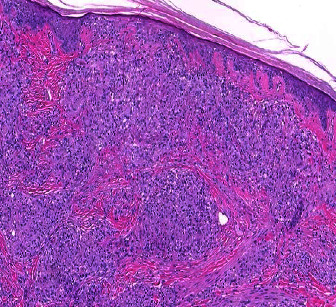
H&E-stained biopsy specimen at 40x magnification.

A woman in her 50s with a history of hand dermatitis, gastroparesis, and achalasia was hospitalized for feeding tube dysfunction. Dermatology was consulted for an itchy and painful rash on her chest and right leg. The patient had been seen for a similar rash on the chest one year prior and was diagnosed with contact dermatitis for which she was treated with triamcinolone 0.1% ointment twice a day with no improvement. A biopsy of the chest had not been performed. She denied any fevers, chills, or recent new lotions or washes. A physical examination revealed an erythematous, scaly patch on her anterior chest surrounding a previous surgical scar (Figure 1) and well-circumscribed, reddish-brown, slightly scaly macules surrounding a large atrophic scar from a prior skin graft on the right leg (Figure 2). A complete blood count with differential and comprehensive metabolic panel was within normal limits. The patient underwent 4 mm punch biopsies of the chest and right leg which revealed scattered, well-formed, non-necrotizing granulomas involving the dermis, composed of epithelioid histiocytes and multinucleated giant cells surrounded by a sparse infiltrate of lymphocytes and histiocytes (Figures 3 and 4). The immunohistochemical staining of the specimen was negative for GMS, Gram, Fite, and PAS stains. A VVG stain did not show fragmented elastic fibers within the lesion. These biopsy results suggested cutaneous sarcoidosis. A CT chest was ordered, which demonstrated extensive hilar lymphadenopathy. This led to a diagnosis of stage II pulmonary sarcoidosis with cutaneous involvement. Of note, an MRI spine was completed during a previous hospitalization which incidentally showed splenomegaly and splenic lesions as well as enlarged periportal and perigastric lymph nodes. These nodes were later biopsied that demonstrated non-necrotizing granulomatous inflammation highly suggestive of sarcoidosis. However, the possibility of sarcoidosis was not explored further at that time, as the patient was lost to follow-up. For this patient, we felt that methotrexate was the best option due to her history of recurrent infections and central line-associated bloodstream infections. She was started on methotrexate 15 mg weekly without complications. Five months later, she endorsed improvement in her lower extremity skin lesions. From a pulmonary standpoint, her disease is stable on methotrexate.

## 3. Discussion

Sarcoidosis is a multisystem granulomatous disease; although cutaneous symptoms are seen in only 25% of patients, the diagnosis is more commonly made based on skin findings rather than pulmonary symptoms [5, 6]. However, cutaneous sarcoidosis can resemble other disorders such as granuloma annulare, leprosy, cutaneous tuberculosis, lichen planus, and lupus pernio [7]. Thus, a biopsy is helpful for diagnosis.

In the case of our patient, her rash was initially diagnosed as contact dermatitis, and she did not follow-up with dermatology as an outpatient even though her rash did not resolve. Once she was readmitted to the hospital a year later, a prompt skin biopsy ultimately led to her pulmonary sarcoidosis diagnosis. This illustrates the importance of having a strong clinical suspicion for sarcoidosis, as this is often necessary to uncover underlying systemic diseases.

For asymptomatic and stable patients, treatment is not always necessary. For those with a skin-limited disease, topical or intralesional corticosteroids are the first line [8]. Patients with symptomatic, severe, or progressive systemic disease are often treated with a combination of systemic glucocorticoids and steroid-sparing therapy such as methotrexate, azathioprine, leflunomide, mycophenolate mofetil, or hydroxychloroquine, or these steroid-sparing treatments may be used alone [8].

In summary, we report a rare case of scar sarcoidosis along with periportal and perigastric lymph node involvement that developed on old scar areas. The skin is the second most common organ affected in sarcoidosis but often the first organ system involved in initial presentation. Prompt cutaneous diagnosis leads to earlier systemic evaluation and potential treatment.

## Data Availability

No data were used to support the study.

## Abbreviations:

GMS: Gömöri methenamine silver
Fite: Fite acid fast
PAS: Periodic acid-Schiff
VVG: Verhoeff–Van Gieson
CT: Computed tomography.
